# Repeated heart rate variability monitoring after myocardial infraction – Cohort profile of the MI-ECG study

**DOI:** 10.1016/j.ijcha.2025.101619

**Published:** 2025-01-26

**Authors:** Teemu Pukkila, Jani Rankinen, Leo-Pekka Lyytikäinen, Niku Oksala, Kjell Nikus, Esa Räsänen, Jussi Hernesniemi

**Affiliations:** a Computational Physics Laboratory, Tampere University, P.O. Box 600 FI-33014 Tampere, Finland; b Faculty of Medicine and Health Technology, Tampere University, Arvo Ylpön katu, 34 FI-33520 Tampere, Finland; c Finnish Cardiovascular Research Center Tampere, Arvo Ylpön katu 34 FI-33520 Tampere, Finland; d Heart Hospital, Tampere University Hospital, Elämänaukio 1 (N building) FI-33520 Tampere, Finland; e Centre for Vascular Surgery and Interventional Radiology, Tampere University Hospital, Arvo Ylpön katu 34 FI-33520 Tampere, Finland

**Keywords:** Heart rate variability, Holter recording, Electrogardiography, Myocardial infarction

## Abstract

The purpose of this study was to monitor heart rate variability (HRV) parameters after myocardial infarction (MI), addressing the ongoing controversy regarding their prognostic value. HRV was measured via Holter monitoring during the acute phase around the time of discharge and again two weeks later during recovery. Our findings show that HRV parameters remained stable during the initial weeks post-MI, indicating that the timing of Holter monitoring in this period is not critical.

Several HRV parameters were significantly correlated with MI type, Killip class, and left ventricular ejection fraction (LVEF), with reduced HRV observed in STEMI patients and those with decompensated heart failure. However, after adjusting for GRACE score and LVEF, the prognostic value of most HRV measures for predicting future cardiac events diminished. Notably, detrended fluctuation analysis DFA1 α_2_ yielded a significant hazard ratio (HR) of 0.79 when adjusted for the GRACE score. However, this significance diminished after adjusting for LVEF (HR = 0.84).

In conclusion, HRV parameters reflect MI severity and correlate with clinical characteristics, but their independent predictive value for future cardiac events is limited when adjusted for established risk factors such as LVEF and GRACE score.

## Highlights

• There are no significant changes in heart rate variability during the initial weeks after myocardial infraction.
• Heart rate variability shows statistically significant differences between myocardial infraction types and severities.
• Detrended fluctuation analysis DFA1 α2 yields a significant hazard ratio of 0.79 when adjusted for the GRACE score.

## 1 Introduction

Myocardial infarction (MI) remains a major global concern, particularly among the aging populations. Its prevalence varies with age, affecting 3.8 % of individuals under the age of 60 years and a staggering 9.5 % of individuals 60 and older. [1] In the United States alone, MI claims over 1 million lives annually. [2] Despite modern advancements in treatment, post-MI mortality rates remain significantly high, with estimates ranging from 7 % to 20 % within the first year following the MI event. [3], [4], [5] More than half of all deaths occur in the initial 30 days following an MI. [6], [7] The initial recovery of the myocardium occurs within first weeks. During this time the risk of arrhythmias is high, and cardiac function is subject to change.

Heart Rate Variability (HRV) is a physiological phenomenon characterized by variations in the time interval between heartbeats. It is essentially measured by analysing the differences in beat-to-beat intervals. HRV serves as an important indicator of the body's ability to adapt to stress and is a significant marker of physiological resilience and behavioural flexibility. This variability reflects an individual's capacity to effectively adjust to changing circumstances. [8], [9] Recently new HRV methods have shown promising results in the prediction of sudden cardiac death (SCD). [10] In the post-MI context of, reduced HRV has been identified as a predictor of mortality. [11], [12], [13], [14] This association arises because reduced HRV indicates diminished adaptability to physiological stressors, which can negatively impact the recovery phase of MI. [9].

Although numerous studies from several decades ago provide observations regarding HRV, its integration into clinical practice to inform patient care remains limited. The main limitations of applying the earlier study findings include the changed treatments and epidemiology, uncertainty around the optimal timing for HRV measurement, and how much these measurements fluctuate during the acute phase. Additionally, it is unclear whether the association between HRV and the risk of severe adverse events is independent of clinical risk factors such as left ventricular ejection fraction (LVEF).

The observational prospective MI-ECG study (Clinical Trials Identifier: NCT03231826) was designed to provide clarity about using continuous ECG monitoring in post MI patients in a modern setting for predicting serious adverse events such as cardiac mortality, sudden cardiac arrests and strokes. In MI-ECG study, a novel dataset of 490 patients was collected with more than 730 long-term Holter recordings conducted either during hospital stay or immediately upon discharge, as well as two weeks after the hospitalization.

## 2 Methods

### 2.1 Study cohort

The MI-ECG study (ClinicalTrials.gov ID NCT03231826) is a prospective clinical observational study in which all consecutive and willing MI patients, who underwent diagnostic coronary angiography at Tampere Heart Hospital were recruited between June 9th of 2017 and January 30th of 2019. MIs were defined according to the ESC criteria for non-ST elevation MI and ST-elevation MI. [15] The Tampere Heart Hospital is the sole provider of specialized cardiac care for emergencies within the region of Pirkanmaa (with a catchment population of over 0.5 million inhabitants). Patients who were in cardiogenic shock at admission, who had otherwise poor functional of mental/cognitive status and were thus unable to give informed consent, or with an estimated life expectancy less than one year estimated by study personnel were deemed ineligible for participation. Written informed consent was obtained from all recruited participants, and the study protocol received approval from the Ethical Committee of the Tampere University Hospital District (ETL R17023). The study adheres to the ethical principles outlined in the Declaration of Helsinki for medical research involving human subjects.

During the recruitment phase, 490 patients were enrolled in the study accounting for 34.0 % of all patients treated for MI (n = 1443) and 10.4 % of all patients undergoing coronary angiography (n = 4713) in the study centre. Several reasons contributed to the non-participation of potentially eligible individuals. These included logistical difficulties, such as patients residing at significant distances from the study centre or those who received treatment and were transferred during the weekend, when recruitment efforts were infeasible. Additionally, the subjective perception of poor health, either by the patient or physician, as well as a lack of interest in participating in the study, also deterred participation.

**Fig. 1:**
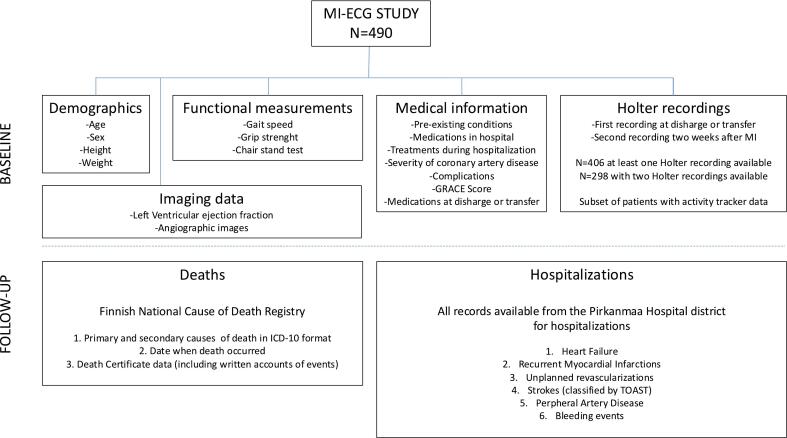
Description of the study population and data sources utilized in this study population.

For this study, we selected patients with at least one technically successful Holter recording, totalling 406 patients with 730 recordings. The selection of patients and collecting data is presented in Fig. 1. Some intended Holter recordings were not completed due to logistical issues, such as the patient being transferred before the recording could begin, or the need for extended in-hospital telemetry recording. Additionally, some patients were not in a suitable clinical condition to start an extra recording.

### 2.2 Holter recording protocol

For each participant, two distinct Holter monitor recordings, each with a duration of at least 24 h, were attempted. The initial recording was scheduled to occur at discharge or as soon as clinically feasible, once regular in-hospital telemetry was no longer necessary. The second recording was scheduled at two weeks following hospital admission for MI. The recordings were conducted using either the GE Seer 12-channel Holter device (GE Healthcare Ltd, United States) or the three channel Bittium Faros 360 device (Bittium Ltd, Finland). The Sampling rate was set to 1024 Hz for the GE Seer device (n = 183 recordings) and a minimum of 500 Hz for the Faros device (n = 535). Accidentally three Faros recordings were made with lower sample rate (n = 2 with 250 Hz and n = 1 with 125 Hz). See Supplementary Table 1 for more details about the recording devices and sampling rates. The GE Seer device was generally preferred for patients with ST-elevation MI. However, no specific criteria governed device selection beyond this. The median interval from hospital admission to the first Holter recording was 3 days with an IQR between 2–5 days. For the second Holter recording, the median interval was 15 days with an IQR of 14 to 17 days. Patients were instructed to maintain their regular daily activities during the monitoring period and were provided with guidance on reinserting the leads to patches should they become disconnected.

**Table 1:** Abbreviations and descriptions of the heart rate variability measures utilized in this study.

**Abbreviation**	**Description**
**Time domain measures**	
Mean RR	Mean value of the RR intervals
SD RR	Standard deviation of the RR intervals
CV RR	Coefficient of variation, defined as standard deviation divided by mean value
RMSSD	Root mean square of successive RR intervals’ differences
pRR20	Percentage of successive RR intervals that differ by more than 20 ms
pRR50	Percentage of successive RR intervals that differ by more than 50 ms
**Frequency domain measures**	
HF	absolute power of the high-frequency band (0.15–0.4 Hz)
LF	absolute power of the low-frequency band (0.04–0.15 Hz)
LF/HF	Ratio of LF-to-HF power
**Poincare plots**	
SD1 RR* SD2 RR	SD perpendicular to the line of identity of the Poincaré plot SD along the line of identity of the Poincaré plot
SD1/SD2 RR	Ratio of SD1 and SD2 measures
**Detrended fluctuation analysis**	
DFA1 α_1_	Short-scale (scales 4–16) scaling exponent of DFA with first-order detrending
DFA2 α_1_	Short-scale (scales 4–16) scaling exponent of DFA with second-order detrending
DFA1 α_2_	Long-scale (scales 16–64) scaling exponent of DFA with first-order detrending
DFA2 α_2_	Long-scale (scales 16–64) scaling exponent of DFA with second-order detrending

*SD1 RR is identical to RMSSD with different constant coefficient so this is only used for calculating SD1/SD2 RR.[40].

### 2.3 Interpretation of Holter recordings during patient recruitment period

The Holter monitor recordings were analyzed by the supervising physicians (JH and LPL) within a week to identify incidents of new-onset atrial fibrillation and sustained ventricular tachycardia episodes. This statement data was recorded as such and systematically documented in the research database. Of the 730 recordings conducted throughout the study, statement data was accessible for 714 instances.

### 2.4 Collection of baseline information

Baseline information was collected prospectively using a standardized patient questionnaire, supplemented by interviews conducted by study personnel. This also included a full disclosure review of all written medical records and electronic health records. The data gathered contained information of patient characteristics, prevalent conditions, prescribed medications at discharge or transfer, and clinical status during hospitalization — covering GRACE score, its individual components and ultrasound measured LVEF. Study personnel also collected details of all invasive medical procedures and diagnostic data in a standardized manner, such as extent of coronary artery disease in coronary angiography. Furthermore, a trained physiotherapist assessed grip strength, 10-meter walking speed, and conducted a chair elevation test to time how long it took for participants to rise ten times from a full sitting position without using their hands.

### 2.5 Follow-up, endpoint definition and adjudication

The follow-up of this study was conducted using comprehensive data accrued from the written medical records of Tampere University Hospital and the national mortality database (Statistics Finland). This data contained all serious adverse events, including all major cardiovascular incidents such as hospitalizations for any cardiovascular or cardiac cause, cardiovascular and cardiac mortality, sudden cardiac arrests and deaths and ischemic strokes. The patient records from Tampere University Hospital provide detailed information of every medical procedure and hospitalization or emergency department visits that required specialized (i.e. secondary or tertiary) health care.

The primary endpoint of this study was the incidence of major cardiac events occurring post-discharge. Major cardiac events were defined as hospitalisations due to acute cardiac causes, such as decompensated heart failure, arrhythmias, unstable angina pectoris or myocardial infarction, as well as deaths due to cardiac causes. There was no loss to follow-up, with complete endpoint data available for all cases. Among those patients who died during the follow-up period, an autopsy was performed in 17 % of cases (11 out of 65 patients). The national mortality database, which includes written accounts of the deaths and the written hospital records can be used in conjunction to classify deaths accurately. [20] This allows for classifying events such as sudden cardiac deaths according to the guidelines of ESC and AHA for sudden cardiac arrests and deaths. [20], [21], [22], [23] More in general, a cardiac death was defined as a death primarily causes by a disease categorized under ICD-10 diagnosis of I11-I59. Cardiovascular death was categorized under ICD-10 diagnoses I10-I99. Hospitalization for any cardiovascular cause was defined as at least one overnight hospitalization due to decompensated heart failure (Killip class > 1 event requiring intravenous diuretics), ischemic stroke, MI, or unstable angina pectoris. Data collection for these endpoints continued beyond patient recruitment until 30th of June 2023, resulting in a median follow-up duration of 5.3 years (IQR 4.9–5.7). The endpoint data will be updated every two years until a maximum of twenty years of follow-up is reached for each patient.

## 3 Statistical methods

### 3.1 R peak detection from the ECG recordings

R peaks are detected from the ECG signal with in-house algorithm (QRS detection specificity 99.5 % and sensitivity 99.6 % with 30 ms threshold for the MIT-BIH Arrhythmia Database[24], [25], [26]) that involves three main steps: I) Define approximate QRS complex locations, II) Perform ECG denoising and baseline wander removal and III) Detect R peaks based on QRS complex morphologies.

First, we determine the approximate QRS complex locations with morphological derivative differences [27], followed by the Shannon energy transform. [28] Detection of signals from multiple leads are then combined using principal component analysis. Approximate R peaks are identified from the roots of the Hilbert transform [28] and approximate QRS complex location is determined as FWHM of the detection signal.

Next, we denoise the ECG and remove the baseline wander with the QRS complexes masked to preserve their morphologies. This process is executed using Complete ensemble empirical mode decomposition with adaptive noise (CEEMDAN). [29], [30].

Following this, we reassess the QRS complex locations on the baseline wander removed signal. Finally, the R peaks are detected based on the QRS morphologies characterized by the number of waves detected and their polarities. [31] More detailed information about the complete algorithm can be found in a Master's thesis. [32].

### 3.2 Data filtering for HRV analysis

Our primary focus in the HRV analysis was on the automatic detection of normal beat-to-beat intervals throughout the entire Holter recording. The RR interval time series was filtered to eliminate unusual intervals. This RR interval cleaning was performed before the data analysis and was thus blinded from the results of the analysis.

First, we excluded RR intervals that were below 200 ms or exceeded 2000 ms, as these extreme values were considered unrealistic for normal beat-to-beat intervals. Secondly, we calculated a rolling median (µ) with a kernel size of 31 beats and removed beats that deviated from the median using the condition 0.75µ < accepted peak < 1.33µ. The thresholds were chosen based on a visual inspection of both filtered and unfiltered time series, independent of the study's outcome. A similar methodology has been previously applied in our HRV studies. [33], [34], [35].

### 3.3 Main exposure variables

Several measures of heart rate variability (HRV) were employed as exposure variables, including time-domain, frequency-domain, Poincaré, and nonlinear methods (refer to Table 1). [9] The transformation from time into frequency domain was achieved with the Lomb-Scargle periodogram, allowing direct calculation of the power spectrum from unevenly sampled time series. [36] Given that the low- and high frequency power (LF and HF) values span multiple orders of magnitude, a base 10 logarithmic transformation was applied to these measures in the hazard models. For nonlinear analysis, we chose detrended fluctuation analysis (DFA). [37] In our DFA calculations we utilized both the conventional short-scale scaling exponents α_1_ (for scales 4–16) and long-scale scaling exponents α_2_ (for scales 16–64). [38] Both exponents were calculated with maximally overlapping windows for better statistical properties [39] and detrending orders 1 and 2. Utilizing maximally overlapping windows and higher detrending order has been previously demonstrated to improve the detection of long QT syndrome patients [34] and the prediction of sudden cardiac death. [10].

### 3.4 Statistical analysis

The association between HRV measures and primary endpoints was assessed with the Fine-Gray subdistributional hazard model, considering the presence of competing risks. The time scale was the duration of the follow-up period. All continuous variables, except for age, were standardized to have a zero mean and unit variance i.e. the hazard ratio reflects the risk change associated with one standard deviation change in the particular measure. For age the hazard ratio corresponds to the risk change associated with a one-year increase in age.

The differences between the initial and subsequent ECG recordings were analysed with Wilcoxon signed-rank test for two matched samples to determine *p*-values. Importantly, only subjects with both recordings were included to allow for a one-to-one comparison of each recording. The impact of changes in heart rate was assessed through a linear mixed-effects model (LMEM), where the measurement served as the dependent variable. Holter (initial vs. subsequent) and the mean RR interval were considered fixed effects, with a random intercept specified for each subject to address within-subject variability. [41] P-values for group comparisons were calculated with Welch’s *t*-test in case of binary variables, while one-way ANOVA was employed otherwise. Correlation coefficients were derived using Pearson's correlation. An unadjusted p-value of 0.05 or less was regarded as statistically significant. The analyses were performed using Python/R-software statistical packages, including pandas, numpy, matplotlib, statsmodels, seaborn and cmprisk.

## 4 Results

The general characteristics of the study population are detailed in Table 2. The average age of participants was 65.8 years (SD 11.2), with two-thirds of the population being men. A small percentage (12.9 %) of the patients experienced decompensated heart failure, classified as Killip class II-III. Of the original 490 participants, 406 (82.9 %) completed at least one Holter recording, and 298 (60.8 %) participants completed both recordings. The median recording duration was 24.2 h, with an interquartile range of 23.9 to 24.9 h.

**Table 2:** Basic statistics of the study population, categorized into three groups: all subjects, subjects with at least one long-term ECG recording, and subjects with two or more long-term ECG recordings. Continuous variables are reported as mean ± standard deviation. Abbreviations: BMI, Body-Mass Index; PCI, Percutaneous Coronary Intervention; CABG, Coronary Artery Bypass Grafting; MI, Myocardial Infarction; TIA, Transient Ischemic Attack.

Variable	All subjects	≥1 long ECG	≥2 long ECG
N (M/F)	490 (347/143)	406 (294/112)	298 (222/76)
Smoking	26.9 % (n = 132)	26.4 % (n = 107)	26.8 % (n = 80)
Ex smoker	22.3 % (n = 112)	24.9 % (n = 101)	23.2 % (n = 69)
Age (years)	65.8 ± 11.2	65.1 ± 11.2	64.5 ± 11.0
Weight (kg)	85.4 ± 18.1	86.2 ± 17.5	86.5 ± 17.5
Height (m)	1.72 ± 0.09	1.73 ± 0.09	1.73 ± 0.09
BMI (kg/m^2^)	28.6 ± 5.4	28.8 ± 5.2	28.7 ± 5.2
GRACE score*	113 ± 26	110 ± 25	109 ± 24
Left ventricular ejection fraction	51 ± 11	52 ± 11	53 ± 10
Previous PCI	15.1 % (n = 74)	15.3 % (n = 62)	15.4 % (n = 46)
Previous CABG	6.9 % (n = 34)	7.4 % (n = 30)	6.4 % (n = 19)
STEMI	46.7 % (n = 229)	45.1 % (n = 183)	41.2 % (n = 123)
History of Heart Failure	13.5 % (n = 66)	10.3 % (n = 42)	8.4 % (n = 25)
Hypertension	62.0 % (n = 304)	61.3 % (n = 249)	61.1 % (n = 182)
Diabetes	26.3 % (n = 129)	24.6 % (n = 100)	22.8 % (n = 68)
History of Stroke or TIA	9.8 % (n = 48)	9.9 % (n = 40)	9.7 % (n = 29)
Prevalent vascular disease	81.6 % (n = 400)	80.8 % (n = 328)	82.6 % (n = 246)
Beta blockers	85.7 % (n = 420)	85.7 % (n = 348)	83.6 % (n = 249)
Killip class I	87.1 % (n = 427)	89.2 % (n = 362)	90.9 % (n = 271)
Killip class II	9.8 % (n = 48)	8.1 % (n = 33)	6.4 % (n = 19)
Killip class III	3.1 % (n = 15)	2.7 % (n = 11)	2.7 % (n = 8)

*GRACE six month mortality[42].

**Killip classification[43].

### 4.1 Comparison of HRV measurements between acute phase and recovery phase

**Fig. 2:**
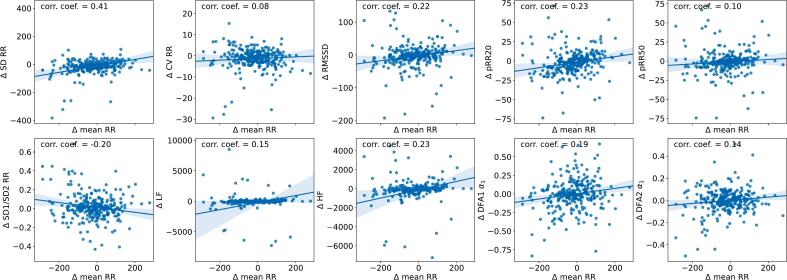
Regression plots showing the relationship between changes in selected HRV parameters and changes in mean heart rate between the acute and recovery phases. Confidence intervals for the regression line were calculated by bootstrapping the samples 1000 times. A few outliers where the heart rate changed by more than 300 ms between the measurements are not shown in the figures but are still included in regression analysis.

Holter recordings from the acute phase and recovery phase showed statistical differences in nearly all time-domain, frequency-domain and Poincaré plot-based HRV measurements with an evident upward trend (Table 3). However, this statistical significance was largely due to the large sample size and variations in mean heart rate. In absolute terms, the differences were quite small when compared to the standard deviation of these parameters. After adjusting for heart rate in the regression analysis using LMEM, only a few HRV measures remained statistically significant. This was mainly caused by changes in overall heart rate which decreased from the acute phase to the recovery phase (Table 3). As illustrated in Fig. 2, the changes in HRV parameters correlate with changes in mean RR interval duration. Most measures exhibit a positive regression line with positive bootstrapped confidence intervals, indicating dependency on heart rate with weak correlations. Some measures, such as SD, show relatively strong correlations despite high intersubject variability. In contrast, DFA measurements, the ratios between SD1 and SD2 of the Poincaré plots, LF and HF remained largely unchanged between acute and recovery phase recordings (Table 3).

**Table 3:** Mean values of heart rate variability measures from the first and second long-term ECG recordings in patients treated for myocardial infarctions. Wilcoxon signed-rank test p-values are presented to evaluate unadjusted differences, while Pearson correlation coefficients show the correlations between measurements. Continuous variables are reported as mean ± standard deviation. Heart rate-adjusted comparisons are analyzed using a linear mixed-effects model (LMEM), with statistically significant p-values (<0.05) highlighted.

Feature	1. Holter	2. Holter	*p*-value	correlation	LME *p*-value
mean RR	927 ± 121	944 ± 139	0.004	0.655	**−**
**SD RR**	**123 ± 48**	**138 ± 64**	**<0.001**	**0.440**	**<0.001**
**CV RR**	**13.3 ± 4.6**	**14.7 ± 5.9**	**<0.001**	**0.508**	**<0.001**
RMSSD	65.1 ± 46.2	68.6 ± 47.7	0.023	0.673	0.234
pRR20	40.4 ± 22.7	42.5 ± 21.9	0.007	0.713	0.120
pRR50	20.2 ± 21.2	21.3 ± 20.6	0.019	0.681	0.152
**SD2 RR**	**166 ± 65**	**188 ± 88**	**<0.001**	**0.419**	**<0.001**
SD1/SD2 RR	0.28 ± 0.18	0.27 ± 0.16	0.093	0.675	0.152
**IQR RR**	**167 ± 79**	**184 ± 95**	**<0.001**	**0.271**	**0.032**
LF	570 ± 1060	880 ± 4190	<0.001	0.157	0.272
HF	747 ± 1150	930 ± 2320	0.030	0.461	0.216
LF/HF	1.34 ± 1.22	1.27 ± 1.07	0.692	0.755	0.213
DFA1 α_1_	0.94 ± 0.28	0.94 ± 0.27	0.914	0.720	0.689
DFA2 α_1_	0.89 ± 0.26	0.89 ± 0.24	0.948	0.718	0.633
DFA1 α_2_	1.05 ± 0.13	1.05 ± 0.13	0.703	0.643	0.780
DFA2 α_2_	1.09 ± 0.22	1.09 ± 0.22	0.861	0.689	0.698

### 4.2 The association between HRV measures and the severity of myocardial infarction

The examination of HRV measures using both acute and recovery phase Holter recordings showed no significant differences, with minor variations primarily attributed to different baseline heart rates. As a result, the association between HRV measures and MI severity was evaluated using the initial Holter recording, which was closest in timing to the measured data on MI severity, associated with LVEF, systolic blood pressure (SBP), GRACE score and peak troponin T (TnT) levels (see Table 4). All HRV measures exhibit weak or modest correlations with all the measures. The strongest correlations were observed between SD1/SD2 RR and LVEF (r = –0.24).

**Table 4:** Correlation between HRV measures and selected continuous clinical parameters, including left ventricular ejection fraction (LVEF), systolic blood pressure (SBP), GRACE score and Troponin T (TnT).

Feature	LVEF	SBP	GRACE score	TnT
mean RR	0.11	−0.02	−0.01	−0.13
SD RR	0.01	−0.03	−0.0	−0.09
CV RR	−0.01	−0.04	0.01	−0.03
RMSSD	−0.16	−0.04	0.18	0.1
pRR20	−0.18	−0.07	0.07	0.08
pRR50	−0.18	−0.04	0.15	0.11
SD2 RR	0.04	−0.03	−0.03	−0.12
SD1/SD2 RR	−0.24	−0.01	0.20	0.18
IQR RR	0.06	−0.03	−0.04	−0.1
LF	−0.08	−0.03	0.08	0.09
HF	−0.14	−0.02	0.18	0.07
LF/HF	0.18	−0.05	−0.2	−0.11
DFA1 α_1_	0.2	−0.02	−0.22	−0.13
DFA2 α_1_	0.19	−0.03	−0.21	−0.13
DFA1 α_2_	0.08	0.02	−0.1	−0.1
DFA2 α_2_	0.11	0.02	−0.16	−0.08

Significantly, numerous HRV parameters were found to be associated with the type of MI (10 out of 16 HRV measures, as shown in Table 5) and severity of heart failure status as indicated by Killip class (8 out of 16 HRV measures, as presented in Table 6). Specifically, patients with STEMI typically demonstrated lower DFA values, with all comparisons showing statistical significance (p < 0.001). They also had higher values for several time domain features, including RMSSD, pRR20, pRR50, and SD1/SD2, all significant at p < 0.05. Furthermore, a similar table for resuscitation is shown in Supplementary Table 2.

**Table 5:** Comparison of heart rate variability (HRV) measures between patients with ST-elevation myocardial infarction (STEMI) and non-ST-elevation myocardial infarction (NSTEMI). P-values are calculated using Welch’s *t*-test, with statistically significant results (p-values < 0.05) highlighted.

	STEMI	NSTEMI	*p*-value
mean RR	917 ± 140	942 ± 120	0.095
SD RR	122 ± 45	127 ± 54	0.349
CV RR	13.3 ± 4.4	13.4 ± 4.9	0.866
**RMSSD**	**80.4 ± 43**	**59.7 ± 47**	**<0.001**
**pRR20**	**46.2 ± 22**	**38.6 ± 22**	**0.002**
**pRR50**	**26.1 ± 21**	**18.0 ± 21**	**<0.001**
SD2 RR	160 ± 62	172 ± 74	0.095
**SD1/SD2 RR**	**0.38 ± 0.21**	**0.25 ± 0.16**	**<0.001**
IQR RR	158 ± 65	172 ± 90	0.108
LF	707 ± 1300	506 ± 820	0.108
**HF**	**947 ± 1100**	**682 ± 1200**	**0.032**
**LF/HF**	**1.03 ± 1.0**	**1.43 ± 1.3**	**0.002**
**DFA1 α_1_**	**0.83 ± 0.28**	**0.98 ± 0.27**	**<0.001**
**DFA2 α_1_**	**0.80 ± 0.24**	**0.92 ± 0.26**	**<0.001**
**DFA1 α_2_**	**1.00 ± 0.15**	**1.06 ± 0.13**	**<0.001**
**DFA2 α_2_**	**1.01 ± 0.23**	**1.11 ± 0.22**	**<0.001**

**Table 6:** P-values from one-way ANOVA comparing different Killip classes. Statistically significant results (p-values < 0.05) are highlighted.

	Killip class			
	I	II	III	*p*-value
mean RR	936 ± 130	923 ± 150	817 ± 130	0.018
SD RR	125 ± 50	134 ± 57	101 ± 37	0.210
CV RR	13.3 ± 4.6	14.6 ± 5.8	12.6 ± 4.2	0.335
**RMSSD**	**66.3 ± 45**	**91.3 ± 52**	**76.8 ± 45**	**0.022**
pRR20	41.1 ± 22	51.0 ± 25	39.7 ± 21	0.083
**pRR50**	**20.4 ± 21**	**31.4 ± 25**	**24.7 ± 21**	**0.031**
SD2 RR	168 ± 69	176 ± 78	131 ± 45	0.207
**SD1/SD2 RR**	**0.29 ± 0.19**	**0.38 ± 0.20**	**0.41 ± 0.15**	**0.014**
IQR RR	167 ± 80	175 ± 81	124 ± 57	0.212
**LF**	**552 ± 980**	**1090 ± 1800**	**463 ± 600**	**0.041**
**HF**	**747 ± 1100**	**1310 ± 1400**	**866 ± 1300**	**0.049**
LF/HF	1.31 ± 1.2	0.91 ± 0.81	0.82 ± 0.8	0.132
**DFA1 α_1_**	**0.93 ± 0.28**	**0.80 ± 0.27**	**0.73 ± 0.24**	**0.007**
**DFA2 α_1_**	**0.88 ± 0.26**	**0.76 ± 0.2**	**0.76 ± 0.22**	**0.031**
DFA1 α_2_	1.04 ± 0.14	1.00 ± 0.14	0.98 ± 0.17	0.194
**DFA2 α_2_**	**1.08 ± 0.23**	**1.01 ± 0.24**	**0.91 ± 0.24**	**0.027**

Consistently, patients classified in higher Killip classes appeared to possess lower DFA values alongside elevated RMSSD and SD1/SD2 values when contrasted with those without signs of decompensated heart failure (Killip class 1). Notably, only one out of sixteen HRV measures displayed a weak yet statistically significant relationship with resuscitation status; patients who underwent resuscitation had lower LF/HF values (p = 0.032).

### 4.3 The association between HRV measures and incidence of cardiac events

Hazard ratios for basic statistical variables and HRV measures were calculated for both the acute and recovery phase Holter recordings. In the study cohort of 347 patients with data from the acute phase Holter recording and clinical variables, cardiac events were observed in 78 patients (22.5 %) during a follow-up period of 5.3 year (IQR 4.9–5.6). Of these patients with cardiac events, 18 had a fatal outcome, translating to a cardiac mortality rate of 5.2 % among all participants. Additionally, nineteen patients died to other causes, representing 5.5 % of the entire study population. See Supplementary material 1 for the recovery phase population. Supplementary Table 3, Table 4 shows statistical comparison of the HRV measures between patients with and without cardiac events, for acute and recovery phase recordings respectively.

The results of the univariate (unadjusted) testing assessing the relationship between clinical variables and HRV measures and the occurrence of cardiac events are presented in Table 7. In summary, fundamental confounding variables such as age, GRACE score and kidney function (eGFR) were identified as significant risk factors associated with the risk of experiencing a cardiac event in both the acute and recovery phase recordings. In addition, LVEF and diabetes were significant risk factors in the acute phase recordings. A noteworthy number of HRV measures (5 out of 16) showed a statistically significant association with the risk of cardiac events in univariate analyses in the acute phase, whereas none showed significance in the recovery phase. Without adjustment, an increase of one standard deviation in time domain, measures (RMSSD and pRR50) and Poincaré plot measures SD1/SD2 RR were associated with approximately a 23–27 % increase in the risk of cardiac events, decreasing to 9–26 % for the non-significant recovery phase recordings. Conversely, a similar increase in both long-scale DFA features was associated with a 26 % reduction in the risk of cardiac events, decreasing to 14 % and 18 % in the recovery phase. In contrast, some of the non-significant measures such as mean RR and LF/HF showed higher risks in the recovery phase than in the acute phase.

**Table 7:** Univariate hazard ratios for cardiac events as the endpoint, with 95 % confidence intervals (CI) provided. Measures with statistically significant p-values (<0.05) are highlighted.

	1. Holter		2. Holter	
Feature	Hazard [95 % CI]	*p*-value	Hazard [95 % CI]	*p*-value
**Clinical variables**				
**Age**	**1.04 ± [1.02**–**1.07]**	**0.002**	**1.04 ± [1.02**–**1.07]**	**0.001**
BMI	0.84 ± [0.65–1.09]	0.181	0.87 ± [0.66–1.13]	0.293
Smoking	0.98 ± [0.75–1.28]	0.870	1.02 ± [0.77–1.35]	0.882
Sex	0.93 ± [0.57–1.52]	0.773	1.10 ± [0.64–1.90]	0.731
History of stroke	1.16 ± [0.59–2.29]	0.662	1.01 ± [0.47–2.18]	0.970
Hypertension	1.08 ± [0.68–1.72]	0.740	1.02 ± [0.63–1.66]	0.933
**eGFR**	**0.67 ± [0.55**–**0.81]**	**<0.001**	**0.62 ± [0.51**–**0.76]**	**<0.001**
**GRACE score**	**1.65 ± [1.32**–**2.08]**	**<0.001**	**1.63 ± [1.27**–**2.08]**	**<0.001**
**LVEF**	**0.71 ± [0.57**–**0.89]**	**0.003**	0.82 ± [0.64–1.06]	0.128
**Diabetes**	**1.90 ± [1.21**–**2.98]**	**0.005**	1.60 ± [0.97–2.64]	0.063
SBP	0.97 ± [0.75–1.25]	0.813	1.05 ± [0.81–1.37]	0.711
DBP	0.88 ± [0.70–1.10]	0.255	0.99 ± [0.77–1.28]	0.935
Beta blockers	1.21 ± [0.61–2.38]	0.581	1.11 ± [0.55–2.26]	0.773
**Heart rate variability measures**				
mean RR	0.91 ± [0.69–1.21]	0.522	0.80 ± [0.61–1.04]	0.098
SD RR	1.11 ± [0.89–1.39]	0.347	0.87 ± [0.65–1.18]	0.372
CV RR	1.14 ± [0.92–1.43]	0.231	0.97 ± [0.75–1.25]	0.797
**RMSSD**	**1.24 ± [1.01**–**1.52]**	**0.042**	1.09 ± [0.87–1.37]	0.452
pRR20	1.16 ± [0.91–1.46]	0.232	1.06 ± [0.81–1.39]	0.664
**pRR50**	**1.23 ± [1.00**–**1.51]**	**0.048**	1.14 ± [0.90–1.45]	0.288
SD2 RR	1.08 ± [0.84–1.38]	0.557	0.84 ± [0.61–1.16]	0.291
**SD1/SD2 RR**	**1.27 ± [1.02**–**1.57]**	**0.030**	1.26 ± [1.00–1.58]	0.050
IQR RR	1.11 ± [0.87–1.42]	0.383	0.89 ± [0.68–1.16]	0.390
LF	1.13 ± [0.89–1.44]	0.313	0.91 ± [0.70–1.18]	0.477
HF	1.22 ± [0.96–1.54]	0.101	1.07 ± [0.84–1.38]	0.576
LF/HF	0.89 ± [0.67–1.19]	0.440	0.77 ± [0.56–1.05]	0.096
DFA1 α_1_	0.83 ± [0.65–1.04]	0.109	0.79 ± [0.62–1.00]	0.053
DFA2 α_1_	0.92 ± [0.74–1.16]	0.497	0.82 ± [0.66–1.03]	0.083
**DFA1 α_2_ **	**0.74 ± [0.60**–**0.92]**	**0.007**	0.86 ± [0.68–1.08]	0.188
**DFA2 α_2_ **	**0.74 ± [0.59**–**0.94]**	**0.014**	0.82 ± [0.64–1.06]	0.134

We calculated multivariable hazard ratios for each statistically significant HRV measure by individually adjusting them with basic demographic variables (age, sex, eGFR and diabetes), as well as LVEF with included demographic variables and GRACE score, which already accounts for age and eGFR. The results are displayed in Table 8, Table 9, Table 10 respectively. Note that a separate multivariable model was calculated for each HRV measure due to their mutual correlations. In multivariable analyses adjusted for conventional risk factors (age, sex, diabetes and eGFR) only DFA1 α_2_ demonstrated significance in relation to the risk of cardiac events. However, rest of the measures were quite close to statistical significancy and in absolute terms did not differ that much from the DFA1 α_2._ Upon adjusting for LVEF, all the measures became statistically non-significant as presented in Table 9. It is important to note that the changes from adjusting solely for basic demographics were minimal since the parameters were initially only barely significant. A similar outcome was observed when adjustments were made for the GRACE score, which is utilized for MI risk assessment; only DFA1 α_2_ persisted as statistically significant independent predictors of cardiac events as detailed in Table 10. Beta blocker medication affects heart rate and also HRV, however in this study population almost everyone has beta blocker medication. After recalculating the multivariable hazard ratios in Table 8, Table 9, Table 10 while also adjusting for beta blockers, the results (not shown) stayed consistent.

**Table 8:** Multivariable hazard ratios with separate model for each HRV parameter, adjusted for basic demographics of age, sex, eGFR and diabetes. 95 % confidence intervals (CI) are provided, and measures with statistically significant p-values (<0.05) are highlighted.

Feature	Hazard ± [95 % CI]	*p-*value
RMSSD	1.15 ± [0.95–1.40]	0.160
pRR50	1.15 ± [0.95–1.39]	0.156
SD1/SD2 RR	1.18 ± [0.96–1.46]	0.123
**DFA1 α**_**2**_	**0.80 ± [0.65**–**0.99]**	**0.036**
DFA2 α_2_	0.82 ± [0.66–1.02]	0.073

**Table 9:** Multivariable hazard ratios with separate model for each HRV parameter, adjusted for left ventricular ejection fraction (LVEF) and basic demographics (age, sex, eGFR, and diabetes). 95 % confidence intervals (CI) are provided, and measures with statistically significant p-values (<0.05) are highlighted.

Feature	Hazard ± [95 % CI]	*p*-value
RMSSD	1.12 ± [0.92–1.36]	0.257
pRR50	1.11 ± [0.92–1.34]	0.273
SD1/SD2 RR	1.10 ± [0.88–1.38]	0.397
DFA1 α_2_	0.84 ± [0.69–1.02]	0.080
DFA2 α_2_	0.85 ± [0.69–1.06]	0.149

**Table 10:** Multivariable hazard ratios with separate model for each HRV parameter, adjusted by GRACE scores. 95 % confidence intervals (CI) are provided, and measures with statistically significant p-values (<0.05) are highlighted.

Feature	Hazard ± [95 % CI]	*p*-value
RMSSD	1.13 ± [0.92–1.38]	0.258
pRR50	1.13 ± [0.92–1.38]	0.241
SD1/SD2 RR	1.16 ± [0.93–1.46]	0.189
**DFA1 α**_**2**_	**0.79 ± [0.64**–**0.97]**	**0.023**
DFA2 α_2_	0.82 ± [0.65–1.03]	0.085

## 5 Discussion

The present study (MI-ECG) was designed to investigate potential changes occurring in long term ECG monitoring after MI and evaluate the prognostic value of the information obtainable from these recordings. Based on our observations, we conclude that HRV parameters do not substantially change in the initial weeks immediately following MI, suggesting that the precise timing of Holter monitoring during this period is not crucial. Additionally, our findings reveal that several HRV features are significantly associated with both the type of suffered MI and heart failure status, with inferior HRV values observed in STEMI patients and those with decompensated heart failure. Finally, we show that several HRV measures are associated with the risk of future cardiac events after MI regardless of traditional risk factors during the acute phase recordings. Adjustment based on the GRACE score showed significance for only DFA1 α_2_ measure and after adjusting for LVEF, none of the HRV measures were statistically significant. However, despite these adjustments for GRACE score and LVEF, many HRV parameters demonstrated similar HR estimates although with wider confidence intervals comparable to those when the analysis was adjusted for traditional risk factors. The recovery phase recording mostly demonstrated similar HR estimates compared to acute phase recording despite not being statistically significant.

Our data analysis revealed some differences in HRV parameters across time-domain, frequency-domain, and Poincaré plot-based measurements between the acute phase (at discharge or upon transfer from a specialized unit) and the recovery phase (two weeks after a myocardial infarction). Although statistically significant, these differences were minor in absolute terms and largely attributed to a decrease in overall heart rate from the first to the second recording. This reduction in heart rate might be due to improved heart failure status or due to changes in medication (including higher titrated doses of betablockers). Regardless of the cause, change in heart rate accounts for most of the variability between HRV measurements made two weeks apart. Supporting this finding, we did observe that DFA measurements remained consistent across both recordings. Since DFA measures are independent of heart rate, they provide a less biased way to assess HRV. One potential explanation for the stability in HRV measurements could be the widespread use of beta-blocker therapy, which may act to stabilize both heart rate and heart rate variability.

Certain HRV parameters derived from the acute phase Holter recording reveal statistically significant differences across various types and severities of MI. This suggests that HRV could be a potential marker for assessing both the severity and type of MI. The HRV measures showed weak correlations with LVEF, SBP and TnT values measured during admission. However, measures such as DFA, RMSSD, and pRR50 displayed significant correlations with the type of MI (NSTEMI vs. STEMI), Killip class, and ST deviation, indicating a relationship between HRV measures and MI severity. However, it is important to note that in our study population the NSTEMI group had 5 years higher mean age (67 vs 62 years), more medications and around 2 times increased mortality (overall, cardiovascular and cardiac mortalities).

Our study highlights that HRV parameters were significantly associated with both the type of MI and heart failure status. Many HRV values are also associated with the risk of cardiac events occurring during follow-up. However, many of the observed associations were not significant after adjusting for either clinical risk factors, for GRACE score or for LVEF. This finding contrasts with past cohorts which associated HRV parameters with adverse cardiac outcomes following MI. [11], [12], [44], [45], [46] These discrepancies might be attributed to substantial advancements in acute MI treatment and the improved survival rates in more recent years. [47] Importantly, our study is based on a contemporary cohort receiving modern MI management, which may provide a more accurate reflection of current clinical outcomes. Some of the differences can also be explained by the variable methodology between previous studies reporting different outcomes and using different strategies to evaluate HRV related risk (for example using extreme cut offs based on different HRV values).

Recent studies have raised expectations that reduced or altered variability in HRV parameters might specifically predict arrhythmic events and SCD following an acute MI with systolic dysfunction. [48], [49], [50], [51] SCD was not explicitly examined as an endpoint in this study due to the currently low number of incidents and resulting insufficient statistical power. Interestingly, in a separate prospective cohort, we have observed an association between DFA2 α_1_ (measured at rest) and the risk of SCD with one SD increase in this specific measure associated with approximately 50 % lower risk. [10] In this population, we observed a weaker association between DFA2 α_1_ and incidence of cardiac events (one SD increase corresponding to 8 % and 18 % lower risk for the acute and recovery phase recordings respectively). These associations were not significant. Due to the differences in the context when HRV was recorded, difference in patient populations and in the endpoints, these results are not directly comparable. Note that the DFA values for this group are lower compared to the control groups reported in other studies, indicating a heightened risk for cardiac events across the entire group [16], [17].

The limitations of the current study include the following: Patients participating in the present study were significantly younger and in better clinical condition when compared to all patients treated for MI. In this select study population the average age of 65.8 years and only a small portion (12.9 %) experiencing decompensated heart failure (Killip class II-III) and patients in cardiogenic shock were not included. The mean age of acute coronary syndrome patients in the same study center (Tays Heart Hospital) between 2007–2018 was 68.9 years and even including unstable angina patients, the occurrence of Killip II-IV class heart failure is 22.1 % [18]. This inherent selection bias, reduces the overall applicability of the results to only patients with good or average clinical condition to begin with. Also, these results are based on 24-hour recordings, during which participants’ daily activities were not controlled. Individuals in better physical condition are likely more active during the day compared to those in poorer condition, who may spend more time resting or bedridden. This difference in activity levels can introduce significant bias in HRV measures that are closely correlated with heart rate. For example, parameters such as RMSSD, pRR20, and pRR50 could be affected by this activity bias. Furthermore, we did not consider patients not undergoing invasive diagnostics (approximately 8 % of all patients in the study center) which have a very poor prognosis [19]. However, among these patients the mortality to other causes such as dementia and related conditions is very high resulting in lower transferability of measured data to clinical prediction of preventable events. Finally, out of the 490 original participants, only 406 (82.9 %) completed at least one Holter recording and 298 (60.8 %) completed two recordings. This lack of replicated recordings for all patients reduces the confidence for some analyses. In the future, with extended follow-up, the limitation caused by small number of events for some specific endpoints, such as SCD, may also be addressed.

## 6 Conclusions

The data from this prospective observational study (MI-ECG) demonstrate that heart rate variability (HRV) measures change minimally, if at all, during the first two weeks following myocardial infarction (MI). Many HRV parameters are significantly associated with MI severity, including correlations with MI type, Killip class, and left ventricular ejection fraction (LVEF). While HRV information shows potential for predicting incident cardiac events, its prognostic value is not fully independent of concurrently measured LVEF. Specifically, detrended fluctuation analysis (DFA) showed promise, but its significance diminished after adjusting for LVEF. Overall, HRV parameters reflect MI severity but may offer limited added value in predicting future cardiac events when traditional metrics like LVEF and GRACE score are already considered.

## Declaration of generative AI and AI-assisted technologies in the writing process

During the preparation of this work the authors used OpenAI API through third-party providers in order to correct grammar and improve readability of the paper. After using this tool/service, the authors reviewed and edited the content as needed and take full responsibility for the content of the published article.

## CRediT authorship contribution statement

**Teemu Pukkila:** Writing – review & editing, Writing – original draft, Visualization, Software, Methodology, Investigation, Funding acquisition, Formal analysis. **Jani Rankinen:** Writing – review & editing, Writing – original draft, Investigation. **Leo-Pekka Lyytikäinen:** Writing – review & editing, Resources, Methodology, Data curation, Conceptualization. **Niku Oksala:** Writing – review & editing, Resources, Methodology, Funding acquisition, Conceptualization. **Kjell Nikus:** Writing – review & editing, Funding acquisition, Conceptualization. **Esa Räsänen:** Writing – review & editing, Supervision, Project administration, Funding acquisition. **Jussi Hernesniemi:** Writing – review & editing, Writing – original draft, Supervision, Project administration, Methodology, Investigation, Funding acquisition, Data curation, Conceptualization.

## Declaration of competing interest

The authors declare the following financial interests/personal relationships which may be considered as potential competing interests: Teemu Pukkila reports financial support was provided by Finnish Cultural Foundation. Teemu Pukkila reports financial support was provided by The Finnish Foundation for Cardiovascular Research. Teemu Pukkila reports financial support was provided by The University of Tampere Foundation. Esa Rasanen reports financial support was provided by Business Finland. Teemu Pykkila reports a relationship with MoniCardi Ltd that includes: board membership and equity or stocks. Esa Rasanen reports a relationship with MoniCardi Ltd that includes: board membership and equity or stocks. Teemu Pukkila has patent Inter-beat interval sequence of heart for estimating condition of subject pending to MoniCardi Ltd. Esa Rasanen has patent Inter-beat interval sequence of heart for estimating condition of subject pending to MoniCardi Ltd. If there are other authors, they declare that they have no known competing financial interests or personal relationships that could have appeared to influence the work reported in this paper.
