# Supplementary material for: Repeated heart rate variability monitoring after myocardial infraction – Cohort profile of the MI-ECG study
Source: Int J Cardiol Heart Vasc. 2025 Jan 26;57:101619. doi: 10.1016/j.ijcha.2025.101619 (PMC11802373; doi:10.1016/j.ijcha.2025.101619)
Supplement: Supplementary Data 1 [file mmc1.docx]

**Supplementary material for manuscript**

**Repeated Heart Rate Variability Monitoring After Myocardial infraction – Cohort profile of the MI-ECG study**

Teemu Pukkila^a*^, Jani Rankinen^b,c,d^,Leo-Pekka Lyytikäinen^b,c,d^, Niku Oksala^b,c,e^, Kjell Nikus^b,c,d^, Esa Räsänen^a^, Jussi Hernesniemi^b,c,d^

^a^Computational Physics Laboratory, Tampere University, P.O. Box 600, FI-33014 Tampere, Finland

^b^Faculty of Medicine and Health Technology, Tampere University, Arvo Ylpön katu 34 FI-33520 Tampere, Finland

^c^Finnish Cardiovascular Research Center Tampere, Arvo Ylpön katu 34 FI-33520 Tampere, Finland

^d^Heart Hospital, Tampere University Hospital, Elämänaukio 1 (N building) FI-33520 Tampere, Finland

^e^Centre for Vascular Surgery and Interventional Radiology, Tampere University Hospital, Arvo Ylpön katu 34 FI-33520 Tampere, Finland

***Correspondence**

Teemu Pukkila

e-mail: [teemu.pukkila@tuni.fi](mailto:teemu.pukkila@tuni.fi)

Computational Physics Laboratory, Tampere University, P.O. Box 600, FI-33014 Tampere, Finland

**Supplementary Tables**

*Supplementary table 1: Number of ECG recording with each device and sampling rates.*

| Device | Sampling rate (Hz) | Number of recordings |
| --- | --- | --- |
| Faros | 125 | 1 |
| Faros | 250 | 2 |
| Faros | 500 | 535 |
| Faros | 1000 | 9 |
| Seer | 1024 | 183 |

*Supplementary table 2: Comparison of heart rate variability measures stratified by resuscitation status during acute coronary syndrome (ACS). P-values are calculated using Welch’s t-test, with statistically significant results highlighted.*

|  | Resuscitation | | |
| --- | --- | --- | --- |
| feature | No (n=339) | Yes (n=8) | *p*-value |
| mean RR | 929±130 | 1050±220 | 0.193 |
| SD RR | 123±47 | 178±130 | 0.296 |
| CV RR | 13.3±4.6 | 16.0±7.3 | 0.369 |
| RMSSD | 67.6±46 | 106.0±59 | 0.134 |
| pRR20 | 41.5±22 | 56.0±19 | 0.090 |
| pRR50 | 21.1±21 | 35.7±23 | 0.141 |
| SD2 RR | 165±64 | 238±180 | 0.320 |
| SD1/SD2 RR | 0.30±0.2 | 0.35±0.16 | 0.428 |
| IQR RR | 163±69 | 271±260 | 0.313 |
| LF | 579±1100 | 1100±1500 | 0.379 |
| HF | 771±1100 | 1780±1900 | 0.200 |
| **LF/HF** | **1.27±1.2** | **0.72±0.54** | **0.032** |
| DFA1 α_1_ | 0.92±0.29 | 0.78±0.24 | 0.161 |
| DFA2 α_1_ | 0.87±0.26 | 0.76±0.17 | 0.127 |
| DFA1 α_2_ | 1.03±0.14 | 1.00±0.12 | 0.504 |
| DFA2 α_2_ | 1.07±0.23 | 0.97±0.21 | 0.239 |

*Supplementary table 3: Comparison of heart rate variability measures stratified by cardiac events during follow-up in acute phase recordings. P-values are calculated using Welch’s t-test, with statistically significant results highlighted.*

|  | Cardiac Event | | |
| --- | --- | --- | --- |
| feature | No (n=X) | Yes (n=X) | *p*-value |
| Mean RR | 936±120 | 922±150 | 0.482 |
| SD RR | 124±46 | 129±65 | 0.487 |
| CV RR | 13.3±4.5 | 13.9±5.4 | 0.330 |
| RMSSD | 65.3±44 | 77.9±53 | 0.061 |
| pRR20 | 41±21 | 44.7±25 | 0.254 |
| pRR50 | 20±20 | 25.6±25 | 0.076 |
| SD2 RR | 167±62 | 171.0±90 | 0.685 |
| **SD1/SD2 RR** | **0.29±0.2** | **0.35±0.23** | **0.033** |
| IQR RR | 166±66 | 173±120 | 0.602 |
| LF | 567±1100 | 709±1100 | 0.333 |
| HF | 721±1100 | 1060±1500 | 0.060 |
| LF/HF | 1.3±1.2 | 1.15±1.2 | 0.330 |
| DFA1 α_1_ | 0.94±0.28 | 0.87±0.29 | 0.069 |
| DFA2 α_1_ | 0.88±0.26 | 0.85±0.25 | 0.366 |
| **DFA1 α_2_** | **1.05±0.14** | **0.99±0.16** | **0.011** |
| **DFA2 α_2_** | **1.09±0.22** | **1.01±0.27** | **0.015** |

*Supplementary table 4: Comparison of heart rate variability measures stratified by cardiac events during follow-up in recovery phase recordings. P-values are calculated using Welch’s t-test, with statistically significant results highlighted.*

|  | Cardiac Event | | |
| --- | --- | --- | --- |
| feature | No (n=X) | Yes (n=X) | *p*-value |
| mean RR | 950±130 | 916±150 | 0.093 |
| SD RR | 141±65 | 132.0±57 | 0.257 |
| CV RR | 14.8±5.9 | 14.5±5.7 | 0.696 |
| RMSSD | 69.7±46 | 75.5±50 | 0.393 |
| pRR20 | 43.7±21 | 45.1±25 | 0.682 |
| pRR50 | 21.8±20 | 25.2±24 | 0.294 |
| SD2 RR | 191±90 | 175±79 | 0.165 |
| SD1/SD2 RR | 0.27±0.2 | 0.33±0.2 | 0.053 |
| IQR RR | 188±100 | 175±81 | 0.274 |
| LF | 917±4400 | 610±990 | 0.318 |
| HF | 933±2400 | 974±1300 | 0.853 |
| **LF/HF** | **1.27±1.0** | **1.01±0.85** | **0.037** |
| **DFA1 α_1_** | **0.94±0.26** | **0.86±0.28** | **0.031** |
| **DFA2 α_1_** | **0.89±0.24** | **0.83±0.21** | **0.046** |
| DFA1 α_2_ | 1.05±0.13 | 1.02±0.15 | 0.161 |
| DFA2 α_2_ | 1.09±0.22 | 1.03±0.27 | 0.110 |

**Supplementary material**

**1. Population of the recovery phase Holter for the study of cardiac events.**

In the study cohort of 328 patients with data from the recovery phase Holter recording and clinical variables, cardiac events were observed in 68 patients (20.7%) during a follow-up period of 5.3 year (IQR 4.8-5.7). Of these patients with cardiac events, 9 (13.2%) had a fatal outcome, translating to a cardiac mortality rate of 2.7% among all participants. Additionally, sixteen patients died to other causes, representing 4.9% of the entire study population.
